# The impact of athletes’ self-identity on employment readiness: the mediating role of professional identity

**DOI:** 10.3389/fpsyg.2025.1729917

**Published:** 2025-12-08

**Authors:** Mengna Zhao, Jiwen Liu, Dongye Lyu

**Affiliations:** 1 School of Education, Beijing Sport University, Beijing, China; 2 School of Politics and Public Administration, Zhengzhou University, Zhengzhou, China; 3 College of Education Sciences, The Hong Kong University of Science and Technology, Guangzhou, China

**Keywords:** athlete career development, self-identity, professional identity, employment readiness, dual career athletes

## Abstract

**Introduction:**

Athletes’ self-identity and professional identity are crucial factors influencing their career development. Against the backdrop of China’s competitive sports system transformation, the employment preparation status of athletes requires urgent attention. This study aims to investigate the internal mechanisms among athletes’ self-identity, professional identity, and employment preparation adequacy.

**Methods:**

A cross-sectional survey design was employed, utilizing a combination of convenience sampling and snowball sampling to administer questionnaires to 160 active and retired athletes. The research instruments included the Self-Identity Scale, Athletes’ Professional Identity Scale, and Employment Preparation Adequacy Scale.

**Results:**

Structural equation modeling analysis revealed that: athletes’ self-identity significantly positively influences their employment preparation adequacy; athletes’ professional identity plays a partial mediating role between self-identity and employment preparation adequacy; Bootstrap testing showed that the mediation effect accounts for 18.84% of the total effect.

**Discussion:**

The study confirms that athletes’ self-identity not only directly affects employment preparation but also exerts indirect influence through professional identity. This provides a theoretical foundation and practical pathways for constructing athlete career support systems centered on the cultivation of psychological capital.

## 1 Introduction

The careers of athletes are marked by distinctive characteristics such as early specialization, high-intensity training systems, and a relatively short competitive lifespan. This unique professional trajectory means that athletes generally face more severe career transition challenges upon retirement compared to the general population. For most athletes, shifting from a focus on competitive sports to achieving success in the workplace is not only a critical turning point in their personal career development but also a complex process involving psychological adaptation, skill transformation, and social reintegration (Gammelsæter and Solenes, 2013). As the need for retirement or career transition grows, athletes often experience a loss of identity and uncertainty in their professional development after leaving the competitive arena, a change that is frequently accompanied by significant psychological challenges and adaptation pressures (Giannone et al., 2017). How athletes’ self-identity and professional identity affect their career transition and employment readiness has become an urgent issue that requires immediate resolution.

Existing research indicates that athletes generally face varying degrees of employment challenges after retirement. These difficulties are reflected not only in declining economic income and scarce job opportunities but also more profoundly in maladaptation to professional roles, difficulties in reconstructing social networks, and the resulting psychological disparities, among other aspects (Turick et al., 2019; Baki et al., 2025). The core research question examines how athletes’ self-identity affects their employment preparation adequacy, and investigates whether professional identity serves as a mediating factor in this relationship. It aims to reveal the psychological mechanisms in athletes’ career transition process—specifically whether self-identity indirectly enhances employment preparation through strengthening professional identity, thereby providing empirical evidence for constructing effective career support systems for athletes.

## 2 Literature review

Existing employment support policies and research perspectives for athletes predominantly focus on the improvement of external objective factors. Governments and sports administrative departments often assist athletes during their transition by providing financial subsidies, optimizing placement policies, and establishing career transition training systems (Hallmann et al., 2019; Jodai and Nogawa, 2012). In academic research, numerous studies also concentrate on external factors such as the improvement of social security systems, the expansion of job markets, and the effectiveness of vocational skill training (Shutova et al., 2019; Gkduman,et al., 2025; Ryan,et al., 2017). While these external interventions are necessary, they have gradually revealed limitations in practice, as they often treat athletes as passive recipients, overlooking the intrinsic psychological motivations and subjectivity that drive individual behavior.

The “dual career” perspective provides a more comprehensive analytical framework for understanding athletes’ employment preparation. “Dual career” refers to the process in which athletes pursue academic education or vocational preparation concurrently with striving for athletic excellence (Stambulova and Wylleman, 2019). Within China’s traditional pyramid-style training model, athletes’ long-term focus on high-intensity training often leads to insufficient academic engagement and lagging professional skill development (Ge and Peng, 2023), placing them at risk of dual career imbalance. Research indicates that athletes’ ability to successfully coordinate sports and academics directly affects the breadth of their self-awareness, flexibility in professional identity, and ultimate employability (Stambulova, 2024). When athletes’ self-worth becomes excessively tied to athletic performance, their professional identity tends to become more exclusive, facing greater challenges during career transition (Brewer et al., 1993). Conversely, athletes who achieve balance in their dual careers typically develop a more integrated self-identity and begin planning for their post-athletic careers earlier, thereby significantly enhancing their level of employment preparation (Knight et al., 2018).

Bandura's (2001) Social Cognitive Theory provides a theoretical foundation for understanding this process. The theory emphasizes the dynamic, interactive relationship between an individual’s internal factors, such as cognition and emotion, and the external environment and behavior. This implies that athletes’ employment preparation behaviors are not only influenced by external opportunities and resources but are also profoundly shaped by their internal psychological capital. Only through the organic integration of external support and internal psychological drivers can athletes’ career development be truly and effectively promoted. Among the various psychological factors influencing athletes’ career development, self-identity plays a fundamental role. Based on Erikson’s psychosocial development theory, self-identity refers to a consistent and coherent self-concept formed through an individual’s exploration of their abilities, values, and goals (Munley, 1975). For athletes, whose identity is highly defined by their athletic role during their developmental years, this singular role identity often leads to severe self-identity crises when facing retirement and transition (Marin-Urquiza et al., 2018). Conversely, a clear and stable self-identity helps athletes maintain psychological continuity during role transitions, integrating their athletic experiences as part—but not the entirety—of their self-narrative, thereby providing intrinsic confidence and motivation for actively exploring career options and developing feasible plans. Athletes with a stronger sense of self-identity are more likely to view their future positively and engage more actively in career exploration behaviors (Monteiro et al., 2021). Based on this, this study put forward the Hypothesis 1: athletes’ level of self-identity can significantly and positively predict the adequacy of their employment preparation.

Meanwhile, athletes’ professional identity refers to the degree to which an individual accepts, values, and becomes emotionally invested in their identity as an athlete, which is also a key variable requiring in-depth exploration. Social Identity Theory of Tajfel and Turner (1979) provides an important theoretical explanation for how individuals construct their self-identity through group membership. Tajfel argues that an individual’s self-concept is largely derived from their sense of belonging to a group. Group membership not only influences how individuals perceive themselves, but also shapes their behavior and attitudes. In the context of athletes, their identity is often closely tied to their performance in the competitive arena, and this sense of identity becomes particularly significant when athletes face career transitions. Tajfel’s theory suggests that when athletes define themselves as “athletes,” this identity influences how they perceive themselves and their future career choices. On one hand, some scholars worry that premature or overly strong identification with the athletic role may limit career perspectives and lead to a more profound sense of identity loss upon retirement (Foster and Huml, 2017; Arnett, 2007). On the other hand, a growing body of research supports an alternative view that positive professional identity can be transformed into valuable human capital (Caza and Creary, 2016). According to Burke’s identity control theory, when individuals strongly identify with their current role, they proactively take actions to maintain and reinforce this identity (Burke, 2004). An athlete who takes pride in their identity is more likely to transfer the embodied capital acquired during their sports career—such as discipline, resilience, and team spirit—to the workplace, and is more inclined to continue their professional life in sports-related fields, thereby engaging in earlier and more systematic employment preparation (Cabrita et al., 2014). Based on this, this study put forward the Hypothesis 2: athletes’ professional identity can significantly and positively predict the adequacy of their employment preparation.

Moreover, there may be an inherent interconnected mechanism between self-identity and athletes’ professional identity. A mutually reinforcing relationship exists between an individual’s overall self-identity and their identity in specific roles (Swann and Bosson, 2010). An athlete with a clear self-awareness and positive self-evaluation is more likely to view their athletic experiences from a healthy and integrated perspective, regarding them as an important component of their self-worth rather than the sole source, thereby developing a higher level of professional identity that is non-exclusive (Cabrita et al., 2014). This enhanced professional identity, rooted in a stable self-identity, may subsequently serve as a specific psychological lever driving their employment preparation behaviors. In other words, it is hypothesized that athletes’ professional identity may play a mediating role in the relationship between self-identity and employment preparation. That is, self-identity not only directly promotes employment preparation but also indirectly influences it by enhancing athletes’ professional identity (Cabrita, et al., 2014). Based on this, this study put forward the Hypothesis 3: athletes’ professional identity plays a mediating role in the relationship between self-identity and the adequacy of employment preparation. This potential mediating pathway has not yet been sufficiently examined in existing research, constituting the core innovation of this study.

In summary, although existing studies have explored certain aspects of self-identity or professional identity individually, research that integrates both within a single framework to systematically examine how they collectively influence athletes’ employment preparation remains scarce. Specifically, questions such as whether “athletes’ professional identity impedes or facilitates employment preparation” and “through which psychological pathways self-identity exerts its influence” urgently require answers through rigorous empirical research in the Chinese context. This study aims to uncover the intrinsic psychological mechanisms that drive athletes to actively plan their career development, thereby contributing to the theoretical discourse on athletes’ career development and providing more targeted scientific evidence for career education and practical support systems for athletes.

## 3 Materials and methods

### 3.1 Participants

This study employed a cross-sectional survey design and utilized a questionnaire method to collect data, aiming to explore the relationships among athletes’ self-identity, professional identity, and the adequacy of employment preparation, as well as the mediating mechanisms involved. A structured questionnaire was used to measure athletes’ self-identity, professional identity, adequacy of employment preparation, and relevant sociodemographic variables. The study participants included both active and retired athletes. A combination of convenience sampling and snowball sampling was used for questionnaire distribution. The questionnaires were primarily distributed through the following channels: (1) collaboration with provincial sports teams to distribute questionnaires collectively to active athletes; (2) contacting eligible retired athletes through retired athletes’ associations and sports institutions; (3) distributing online questionnaires via online communities for athletes on social media platforms. A total of 175 questionnaires were collected. After excluding invalid responses with overly short completion times or clearly patterned answers (e.g., all options identical), 160 valid questionnaires were retained, resulting in an effective response rate of 91.4% (Table 1).

**Table 1:** Demographics of the participants (*N* = 160).

Item	Option	Frequency	Percentage (%)
Gender	Male	71	44.4
	Female	89	55.6
Age	Under 20 years	67	41.9
	20–30 years	75	46.9
	30–40 years	18	11.3
Education Level	Junior high school or below	32	20.0
	High school	24	15.0
	Bachelor’s degree or associate degree	70	43.8
	Postgraduate	34	21.3
Years Engaged in Specialized Sport	Less than 3 years	6	3.8
	3–5 years	11	6.9
	5–10 years	55	34.4
	More than 10 years	88	55.0
Registered with Specialized Sports Association	Yes	122	76.3
	No	38	23.8
Current Athlete Grade	International elite player	8	5.0
	Elite player	55	34.4
	First-grade Athlete	58	36.3
	Second-grade Athlete	22	13.8
	Third-grade Athlete	4	2.5
	No Grade	13	8.1
Current Professional Status	Active	79	49.4
	Imminent retirement	10	6.3
	Retired	71	44.4

### 3.2 Measures

To examine the impact of athletes’ self-identity on the adequacy of their employment preparation and analyze the mediating role of athletes’ professional identity in this process, this study employed a combination of self-developed questionnaires and standardized scales. The instruments covered core variables including career choice readiness, self-identity, and athletes’ professional identity. Specifically, the research tools consisted of the following three components: the Career Choice Readiness Scale, the Self-Identity Scale, and the Athletes’ Professional Identity Scale. The design of these scales considered both the requirements for quantitative analysis and ensured the validity and reliability of the questionnaires.

#### 3.2.1 Career choice readiness scale

Drawing on the scale design by Chen (2017) and incorporating contextual adaptations based on the career development characteristics of athletes in China, a refined scale comprising 7 items was ultimately developed. The scale employs a Likert 5-point scoring method (1 = “Strongly Agree” to 5 = “Strongly Disagree”), including positive items such as “I have a clear career development plan” and “During my athletic career, I have enhanced relevant capabilities through learning for future career choices,” as well as reverse-scored items like “I am still uncertain about my future job competence” and “Career planning is unnecessary due to the unpredictability of the employment environment.” Prior to data analysis, reverse-scored items were appropriately recoded. The total career choice readiness score was calculated by summing the scores of all items after appropriate recoding, with higher scores indicating better employment preparation among athletes. In the sample of this study, the scale demonstrated good internal consistency, with a Cronbach’s α coefficient of 0.766.

#### 3.2.2 Self-identity scale

The Self-Identity Scale, developed by Ochse and Plug (1986) based on Erikson’s identity theory, was adopted. This scale consists of 19 items and employs a Likert 4-point scoring system (1 = “Strongly Disagree” to 4 = “Strongly Agree”). It covers core dimensions measuring the unity and stability of an individual’s self-concept, including items such as “I know how I should live my life” and “I am proud of who I have become.” Among these, 9 items are reverse-scored. The scale demonstrated ideal reliability in this study, with a Cronbach’s α coefficient of 0.812.

#### 3.2.3 Athletes’ professional identity scale

The Athletes’ Professional Identity Scale, developed by Guan (2017), was used to assess the emotional commitment and internalization of values associated with athletes’ professional identity. This tool comprises 19 items and adopts a Likert 5-point scoring system (1 = “Strongly Disagree” to 5 = “Strongly Agree”). Key measurement items include “I am suited to be an athlete” and “Being an athlete allows me to realize my life’s value,” with one item being reverse-scored. Under the conditions of this study, the scale exhibited good measurement reliability, with a Cronbach’s α coefficient of 0.803.

### 3.3 Data analysis

Data collection was conducted through a combination of online questionnaires and paper-based questionnaires to ensure broad coverage of athletes from diverse backgrounds. After data collection, SPSS 26.0 software was used for analysis, primarily including reliability analysis, validity testing, Pearson correlation analysis, regression analysis, and mediation effect analysis. The study employed the Bootstrap method (Davison and Hinkley, 1997) to test the mediating effect of professional identity between self-identity and employment preparation, further validating the pathway through which professional identity influences employment preparation.

## 4 Result

### 4.1 Reliability and validity tests and common method bias test

Prior to conducting hypothesis testing, the reliability and validity of the core variable measurement tools used in the study were evaluated. The Cronbach’s α coefficients for the Career Choice Readiness, Self-Identity, and Athletes’ Professional Identity scales were 0.766, 0.812, and 0.803, respectively, all exceeding the acceptable threshold of 0.70. This indicates that each scale demonstrated good internal consistency reliability within the study sample. Further exploratory factor analysis (EFA) was conducted to assess structural validity. The results showed a KMO value of 0.842, and Bartlett’s test of sphericity was significant (χ^2^ = 1852.36, *p* < 0.001). The cumulative variance explained was 68.5%, and all items demonstrated factor loadings exceeding 0.45 on their respective factors, indicating good structural validity of the questionnaire. To control for common method bias, Harman’s single-factor test was employed. The unrotated factor analysis extracted three factors with eigenvalues greater than 1, with the first common factor explaining 28.7% of the variance, which is below the critical threshold of 40%. This suggests that common method bias did not pose a significant issue in this study.

### 4.2 Correlation analysis of athletes’ self-identity, professional identity, and employment preparation adequacy

Descriptive statistics and Pearson correlation analysis were conducted for the core variables, with the results presented in Table 2. The mean score for self-identity was 62.45 (SD = 8.73), for athletes’ professional identity was 71.28 (SD = 9.65), and for employment preparation adequacy was 24.32 (SD = 5.18). The correlation analysis revealed a significant positive correlation between self-identity and employment preparation adequacy (r = 0.55, *p* < 0.01). Similarly, a significant positive correlation was observed between self-identity and athletes’ professional identity (r = 0.40, p < 0.01). Additionally, athletes’ professional identity and employment preparation adequacy also showed a significant positive correlation (r = 0.45, p < 0.01). These significant positive correlations lay the foundation for further in-depth analysis of the causal relationships among the variables.

**Table 2:** Correlation tests of core variables.

Measure	Mean	Standard deviation	1	2	3
Self-identity	2.90	0.36	1.00		
Athlete professional identity	3.96	0.60	0.40	1.00	
Employment preparation adequacy	3.62	0.67	0.55	0.45	1.00

Correlation is significant at the 0.01 level (2-tailed).

### 4.3 The relationship between athletes’ self-identity, professional identity, and employment preparation adequacy

To further investigate the relationships between variables, hierarchical regression analysis was conducted to examine the predictive effects of self-identity and athletes’ professional identity on employment preparation adequacy. In the regression analysis, control variables (gender, age, education level) were entered in the first step, followed by the two core independent variables (self-identity and athletes’ professional identity) in the second step. The Variance Inflation Factor (VIF) values ranged from 1.12 to 2.45, well below the critical threshold of 5, indicating no serious multicollinearity issues. The control variables explained 18.2% of the total variance in employment preparation adequacy (*F* = 5.32, *p* < 0.001). After adding self-identity and athletes’ professional identity, the model’s explanatory power significantly improved, with ΔR^2^ = 0.245, F-change = 25.67, p < 0.001, bringing the total explained variance to 42.7%. Both self-identity (β = 0.42, p < 0.001) and athletes’ professional identity (β = 0.25, *p* < 0.01) demonstrated significant positive predictive effects on employment preparation adequacy.

To further investigate the influence process and mechanism among athletes’ employment preparation adequacy, self-identity, and professional identity, this section employs a mediation effect model to examine whether athletes’ professional identity demonstrates a significant mediating effect in the relationship between self-identity perception and employment preparation adequacy. Table 3 presents the results of the Bootstrap method test. The regression results indicate that the total effect coefficient of self-identity on employment preparation adequacy is 0.9752, and its 95% confidence interval does not include zero, suggesting a significant total effect.

**Table 3:** Mediation effect analysis of athletes’ professional identity.

Effect type	Influence path	Effect Coefficient	SE/BootSE	95%Confidence Interval
Total effect of X on Y	Self-identity → Employment preparation adequacy	0.9752	0.1229	(0.7322, 1.22)
Direct effect of X on Y	Self-identity → Employment preparation adequacy	0.7914	0.1279	(0.5386, 1.04)
Indirect effect(s) of X on Y:	Self-identity → Athlete professional identity → Employment preparation adequacy	0.1838	0.0698	(0.0659, 0.3382)

To further verify whether the direct effect of the independent variable (self-identity) on the dependent variable (employment preparation adequacy) is significant, regression results show that after controlling for the mediating effect of athletes’ professional identity, the direct effect coefficient is 0.7914 with a 95% confidence interval of (0.5386, 1.04). This indicates that the direct effect of the independent variable (self-identity) on the dependent variable (employment preparation adequacy) remains significant after controlling for the mediating variable. Furthermore, to examine whether the mediating effect of athletes’ professional identity is significant in the relationship between self-identity and employment preparation adequacy, regression results reveal a coefficient of 0.1838 with a 95% confidence interval that does not include zero. Thus, at the 0.05 significance level, the mediating effect of athletes’ professional identity is statistically significant.

**Figure 1:**
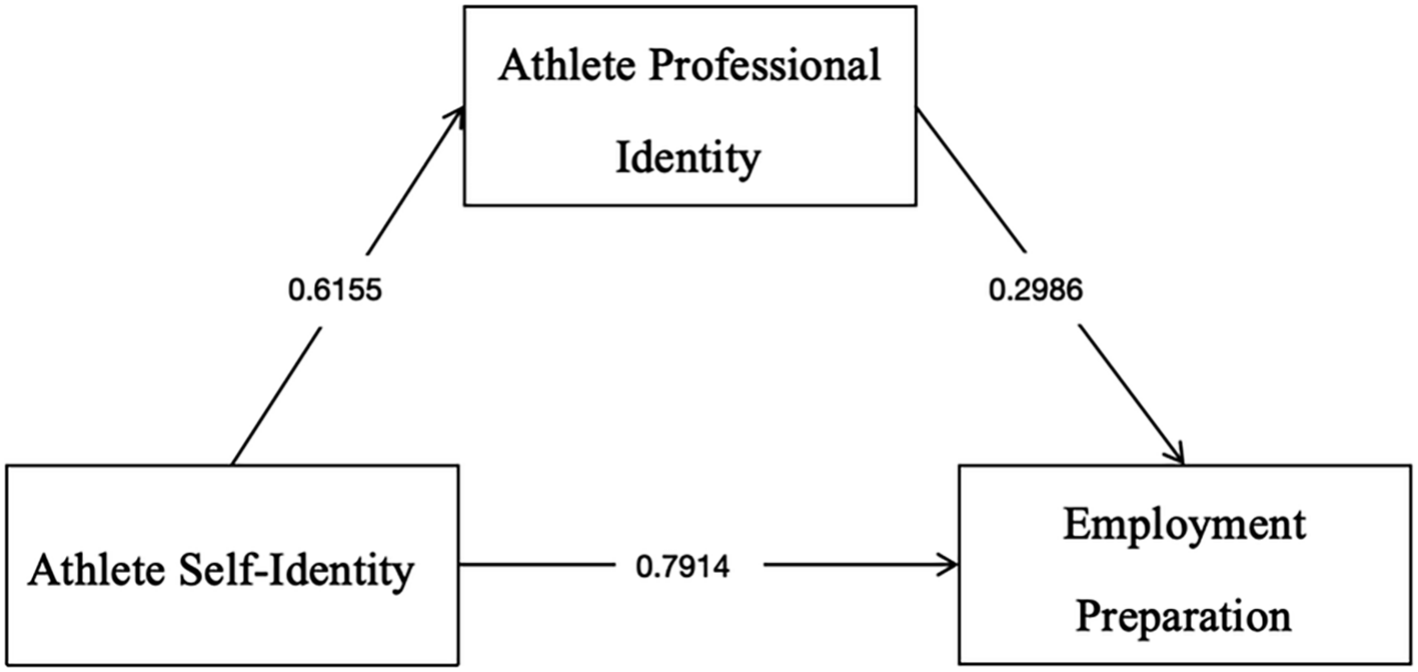
Schematic diagram of the mediating effect.

In summary, athletes’ professional identity plays a significant mediating role in the relationship between self-identity and employment preparation adequacy. Specifically, self-identity influences employment preparation adequacy through two distinct pathways: first, through a direct effect of the independent variable (self-identity), and second, through an indirect effect mediated by athletes’ professional identity. The results further indicate that athletes’ professional identity functions as a partial mediator, with the indirect effect accounting for 18.84% of the total effect (as illustrated in Figure 1).

## 5 Discussion

### 5.1 The positive impact of athletes’ self-identity on employment preparation adequacy

This study verifies the significant positive impact of self-identity on athletes’ employment preparation adequacy. This finding aligns with the key idea of social cognitive theory, which emphasizes the decisive role of individual cognitive factors in behavior choice and preparation. Specifically, athletes with higher levels of self-identity typically have a clearer and more stable self-cognitive framework, including an understanding of their abilities, values, and future developmental directions. This cognitive certainty provides them with the necessary psychological resources and intrinsic motivation, prompting them to more actively explore career paths, develop feasible career plans, and persist in implementing these plans.

Self-identity, as an individual’s sense of acceptance and affiliation with their traits, values, roles, and social identity, plays a crucial role in athletes’ career preparation. Research has shown that strong self-identity not only helps athletes maintain focus and motivation during their sports careers, but also helps them smoothly transition to a new professional identity after retirement (Lally, 2007). Especially at the critical stage of retirement, athletes often face significant challenges in identity transformation. If their identity is overly dependent on the “athlete” role, they may experience an identity crisis, which can affect the continuity of their career (Haslam C. et al., 2024). Therefore, enhancing athletes’ self-identity, especially during career transitions, is an important pathway to improving their employment preparation adequacy.

This finding resonates with the research by Horne et al. (2025) on self-identity in career development. They point out that the strength of an individual’s self-identity, especially during identity transition periods, plays a crucial role in career adaptation and successful transformation. Athletes’ self-identity not only affects their engagement and investment in their current profession, but also influences their preparation for future careers. When athletes have stronger self-identity, they are more likely to actively prepare for jobs outside the sports field, rather than relying solely on the experience and skills from their athletic careers. Therefore, self-identity, as a psychological resource, can stimulate athletes’ motivation to seek new careers after retirement and help them be more proactive in preparing for this process.

Further research indicates that athletes with strong self-identity are more likely to integrate and identify with the experiences and skills accumulated during their sports careers, thus transforming them into new career capital when facing career transitions. This transformation not only helps athletes maintain psychological continuity but also enhances their confidence, making them more relaxed when entering new career fields (Lent et al., 1996; Cosh, 2021). Therefore, enhancing athletes’ self-identity not only helps them maintain high performance during their athletic careers but also lays a solid psychological foundation for their post-retirement career development.

Overall, there is a significant positive relationship between the strength of athletes’ self-identity and their employment preparation. Strengthening self-identity helps athletes more clearly recognize their potential in future careers, thus preparing them well for career transitions. Policymakers and athlete support organizations should focus on this aspect of psychological development, particularly during career transitions, by systematically helping athletes strengthen their self-identity and supporting their smooth transition into the workforce.

### 5.2 The mediating effect of athletes’ professional identity

This study further explores the mediating effect of athletes’ professional identity between self-identity and employment preparation adequacy. Through structural equation modeling and Bootstrap method analysis, we found that professional identity plays a significant mediating role in the influence of self-identity on employment preparation adequacy. This finding not only confirms the key role of professional identity in athletes’ career transitions but also provides a new theoretical perspective for understanding how athletes can enhance their career preparation through self-identity.

First, professional identity, as a part of athletes’ self-identity, reflects their identification with and emotional investment in the “athlete” role. It is not just an athlete’s self-definition of their sports career, but also includes emotional attachment and a sense of responsibility toward their professional identity (Foster and Huml, 2017). Our study shows that athletes with a strong sense of professional identity are better able to successfully transition after retirement, actively seeking career opportunities related to sports or completely different fields. Therefore, the strength of professional identity directly influences athletes’ readiness and adaptability to enter new career fields after retirement. Specifically, the stronger the athletes’ identification with their “athlete” role, the more confidently they can apply their experience and skills to face new professional challenges during the transition.

Moreover, the research results indicate that the mediating effect of professional identity between self-identity and employment preparation adequacy is a partial mediation. This means that although self-identity promotes career preparation by directly influencing employment readiness, the enhancement of professional identity plays an indispensable bridging role in this process. While self-identity positively affects athletes’ career preparation, much of this impact is realized through strengthening athletes’ professional identity. This mediating effect suggests that, in addition to enhancing self-identity, athletes should focus on cultivating their professional identity during the retirement phase, particularly in how to transform the value of their “athlete” role into new professional capital.

We found a positive interactive relationship between athletes’ self-identity and professional identity, meaning that strong self-identity helps enhance professional identity, thereby further promoting employment preparation. Self-identity provides athletes with intrinsic motivation and psychological support, making them more determined and confident when facing the pressures of career transition. Especially during retirement, athletes’ professional identity can enhance their sense of identification with new roles in various ways, such as by redefining the “athlete” identity and transforming it into an advantage for career development (Zhang et al., 2025). For example, athletes can view the skills they accumulated in the competitive arena, such as teamwork, leadership, and time management, as important additions to their future careers, strengthening their identification and responsibility toward new professional roles, thus effectively preparing for entry into non-sporting career fields.

This finding aligns with existing literature, which highlights that athletes’ professional identity not only helps them maintain focus during their sports careers but also provides support during the transition to post-retirement careers (Caza and Creary, 2016; Yao, 2018). For example, when athletes can see the connection between their athletic careers and other career developments and believe that their “athlete” identity extends beyond the sports arena to other fields, their career transitions become smoother.

At the same time, this finding offers practical insights into athlete career transitions. Athletes’ professional identity is not necessarily tied to the end of their sports career but can continue to play a role through psychological adjustment and training after retirement. This means that policymakers and athlete support organizations, in assisting athletes with career transitions, should not only focus on athletes’ self-identity but also place more emphasis on cultivating their professional identity to help them redefine their professional roles. Providing career planning training, psychological support, and role identity reconstruction services can effectively enhance athletes’ professional identity, thereby improving their chances of successful career transition.

By verifying the mediating effect of professional identity between self-identity and employment preparation, this study deepens our understanding of the psychological mechanisms behind athletes’ career transitions and provides a new perspective for future research. Future studies could further explore how different dimensions of professional identity have different effects across various athlete groups, especially between athletes from different sports disciplines and competitive levels. Additionally, considering cultural backgrounds and social support differences, researchers could explore how athletes’ professional identity develops across different countries and regions and how it influences their career adaptation and transition in a globalized context.

The mediating role of professional identity between self-identity and employment preparation highlights the psychological adjustment process athletes undergo during career transitions. Enhancing professional identity provides athletes with stronger support for career transformation, helping them smoothly transition to post-retirement careers. Therefore, future career support systems should focus on cultivating professional identity and provide comprehensive support for athletes’ career transitions.

## 6 Limitations and future research

The deficiencies in this study included the following: First, the sample was characterized by a highly youthful age structure and a predominance of elite athletes, which limits the generalizability of the findings. Future studies should include athletes from diverse career stages, competitive levels, and sports disciplines to test the model’s broader applicability. Second, the cross-sectional research design precludes definitive conclusions about causal relationships between variables. Subsequent research could employ longitudinal tracking designs or intervention experiments to uncover dynamic causal links. Finally, potential biases inherent in self-reported data and the limited scope of research variables should be addressed. It is recommended that future studies incorporate multi-source data (e.g., peer evaluations, objective behavioral indicators) and introduce more diverse variables, such as social support and career decision-making self-efficacy, to construct a more comprehensive explanatory model for athletes’ career development.

## 7 Implications

By validating the partial mediating role of athletes’ professional identity, this study deepens the understanding of the psychological mechanisms underlying athletes’career development. The findings challenge the traditional view that professional identity is simply a barrier during career transition. Instead, it reveals the intrinsic mechanism through which professional identity, when built on a solid foundation of self-identity, can be transformed into a positive psychological resource. This offers a new perspective for constructing a more dialectical and developmental theory of athlete identity, emphasizing that future theoretical frameworks should focus on the synergistic development of self-identity and professional identity rather than treating them as opposing forces.

The results of this study provide clear directions for optimizing the career support system for athletes. First, psychological development should be integrated as a core component of career support. This can be achieved through initiatives such as self-exploration workshops and career narrative counseling to systematically enhance athletes’self-identity. Second, targeted intervention programs should be designed to help athletes develop a healthy professional identity, with a focus on guiding them to recognize the value of transferable skills cultivated through their athletic experiences—such as resilience and teamwork—in the workplace. Furthermore, a stratified support strategy should be implemented: young athletes should receive early career planning guidance, while those with lower educational qualifications should be provided with accessible pathways for academic advancement and vocational skills training. Finally, policy-making should focus on creating an institutional environment that enables athletes to transform their specialized capital into career advantages. For instance, roles such as school coaches could be designed to fully leverage athletes’ professional expertise, establishing more inclusive career transition pathways.

## 8 Conclusion

The empirical analysis of this study confirms that athletes’ self-identity not only directly enhances the adequacy of their employment preparation but also generates indirect positive effects through the key psychological pathway of strengthening professional identity. This finding reveals the core driving role of intrinsic psychological factors in athletes’ career transitions, extending the focus of career development discussions from external support to the cultivation of internal psychological resources. The research emphasizes that when constructing employment support systems for athletes, paramount importance should be placed on building their psychological capital by reinforcing self-identity and fostering a healthy professional identity to stimulate endogenous motivation for career development. Meanwhile, the study’s identified limitations regarding sample representativeness and causal inference point to future research directions employing longitudinal designs and more diverse samples, thereby providing ongoing scientific evidence for developing more comprehensive and effective models to support athletes’ career transitions.

## Data Availability

The raw data supporting the conclusions of this article will be made available by the authors, without undue reservation.
